# Substance abuse in pregnant women. Experiences from a special child welfare clinic in Norway

**DOI:** 10.1186/1471-2458-7-322

**Published:** 2007-11-11

**Authors:** Bjørg Hjerkinn, Morten Lindbæk, Elin Olaug Rosvold

**Affiliations:** 1 Addiction Unit/Research Unit, Sørlandet Hospital, Kristiansand, Norway; 2 Institute of General Practice and Community Medicine, Faculty of Medicine, University of Oslo, Norway

## Abstract

**Background:**

Substance abuse during pregnancy may harm the foetus and can cause neonatal abstinence syndrome. Exposure to alcohol and other substances can influence the child for the rest of its life. A special child welfare clinic was set up in 1994 in Kristiansand, Norway, targeting pregnant women with substance abuse problems in the county of Vest-Agder. Pregnancy is not an indication for opioid replacement therapy in Norway, and one of the clinic's aims was to support the drug dependent women through their pregnancy without any replacements. The object of this paper is to describe concurrent health and social problems, as well as the predictors for stopping drug abuse, in the clinic's user group.

**Methods:**

Retrospective cohort study. Data was gathered from the medical records of all 102 women seen in the clinic in the period between 1992 and 2002. The study includes 59 out of 60 women that were followed until their children were two years old or placed in alternative care, and a comparison group of twice the size. Both groups were presented with a questionnaire concerning both the pregnancy and health and socio-economic issues.

**Results:**

Four (4.5 percent) of the women that completed their pregnancies did not manage to reduce their substance abuse. All the others reduced their substance abuse considerably. The odds ratio for stopping substance abuse within the first trimester was significantly associated with stopping smoking (O.R. 9.7) or being victims of rape (O.R. 5.3).

**Conclusion:**

A low cost and low threshold initiative organised as a child welfare clinic may support women with substance abuse problems in their efforts to stop or reduce their substance abuse during pregnancy.

## Background

Alcohol and other substances taken by pregnant women can harm the unborn baby [1-3]. Pregnant women who continue their substance abuse often give birth prematurely, the infants are often small for their gestational age, and they have more perinatal incidents. Despite evidence for a wide range of negative consequences for the pregnant women and their children, specific programs addressing this group did not begin appearing until the late 1980ies [4]. Female alcoholics and substance abusers have been met with anger and blame, which led to a lack of treatment services. In recent years, models of treatment have been developed [5,6], which have proved to be clinically and economically effective [7-9].

A special child welfare clinic (SCWC) was set up in the city of Kristiansand in 1994 to work with substance abusing pregnant women and mothers with young children, with the aim of helping these women stop their substance abuse without replacement therapy [10]. Pregnancy is not an indication for opioid replacement therapy in Norway. The services offered by the SCWC focused both on the pregnancy and the substance abuse, always with a main focus on the wellbeing of the foetus or child. As pregnancy is called a window of opportunity for substance abusing women to change their way of life, the main objectives of the SCWC were twofold. Firstly, the clinic wanted to establish contact with all pregnant women that were substance abusers or who had recently stopped. Secondly, the clinic intended to support and motivate the women to enable them to end their substance abuse as early as possible in pregnancy in order to prevent their children from being harmed by the substances.

The Special Child Welfare Clinic in Kristiansand is a low threshold initiative organised within the primary health care system as a child welfare clinic reinforced with extra staff. The staff consists of a midwife, a community nurse, a social worker, and a general practitioner, and is specially trained in topics related to substance abuse, addiction and psychiatry.

**Figure 1:**
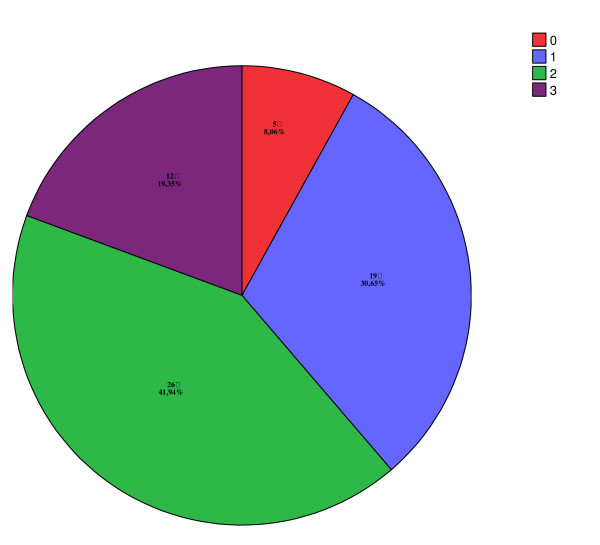
Total number of contacts with other initiatives than SCWC in pregnancy.

As the SCWC is a low threshold and voluntarily initiative, the pregnant women do not need referrals, but the counselling initiatives in the community, the public health service, and others have referred most of the users to the clinic. From the very beginning the clinic has made an effort to offer individual service to the users. Appointments were made as often as once a week, and it was considered important to register and help with the users' special needs concerning housing, economy and necessary health service as soon as possible. We also made social network maps to clarify and cooperate with the human resources close to the users. In addition, we conducted normal pregnancy checkups in close collaboration with the regular general practitioner and the hospital, and made contracts for the frequency with which the user was to provide urine specimens. If the user had a regular general practitioner, we cooperated with him/her about medical issues and pregnancy checkups. If the user did not have a regular general practitioner, she was assigned the doctor at the SCWC. If a user did not keep her appointment, we contacted her by phone or paid a visit to her home in order to set up a new appointment very soon. For all the users we tried to set up network groups consisting of the user, her relatives and the necessary professionals. These groups then met regularly to consider the further progression of the treatment. Since the clinic's inception in 1994, staff turnover has been very low. The routines have also remained relatively unchanged, with the exception that we have tried to increase the frequency of urine specimens. The service is free of charge, as it is for all pregnant women in Norway. We consider this model to be low cost, as the need for resources to hire and train new staff has been low, and the resources required to meet the special needs of each user were provided from existing resources (Figure 1). The role of the SCWC is merely to be the hub of the wheel.

The object of this paper is to describe the socioeconomic status and concurrent health and social problems among the users of the SCWC. Furthermore, we want to explore the changes in substance abuse during pregnancy, the impact of service utilization, and finally, predictors for stopping substance abuse during pregnancy.

## Methods

During the period between 1994 and 2002, 102 pregnant, substance abusing women came through the SCWC. At entry, they had abused substances to such a degree that they had dropped out from school or work. They were all known by the substance abuse counselling or the health care systems in the community to have serious substance abuse problems, and their substance abuse was the main reason for referral to SCWC when they got pregnant.

**Figure 2:**
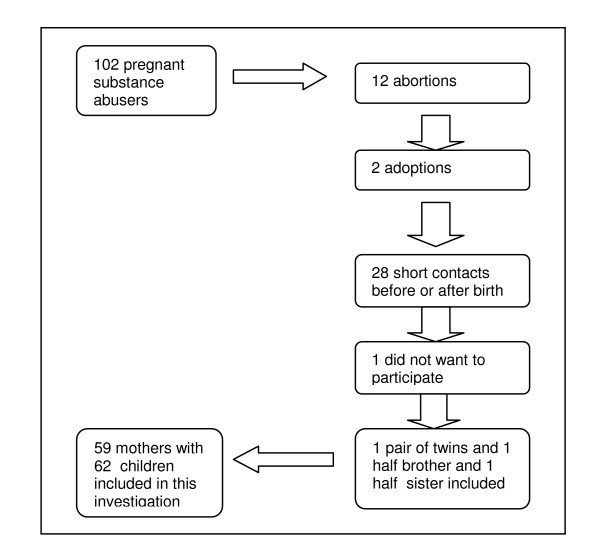
Flow chart of the users of Special Child Welfare Clinic in Kristiansand, Norway in 1994–2002.

Thirteen of the 102 women had abortions, eight spontaneous and five induced. These have been excluded from the further study of substance abuse in pregnancy, along with the mothers of two children who were adopted and 27 women who only had brief contact with SCWC while living temporarily in Kristiansand, serving time in prison, or staying in women's crisis centres. The medical records at SCWC revealed no difference in the group that only had brief contact with SCWC compared to the group that continued treatment at the clinic in terms of substance abuse and socio-economic issues. One mother gave birth to three children and eight mothers gave birth to two children during the period, one of which had twins. Of these pregnancies, only the first is included in the study. In all, 60 mothers kept in contact with SCWC from pregnancy until the child was two years of age or placed in alternative care. In 2004 they were asked to be part of this follow-up study. One mother declined to participate, giving a total of 59 mothers in the study (Figure 2).

We used the diagnosis register of the regional hospital to analyse how large a percentage of the substance abusing pregnant women in the region were admitted to the SCWC. The diagnosis indicating drug use is included in the discharge summary if the urine test from the mother and/or child was positive for illicit drugs. Urine specimens are taken if the mother is suspected of abusing illicit substances.

A comparison group consisting of 169 parents and their children were selected at random from a child welfare clinic in the same building as the SCWC and from two schools in Kristiansand. This group represents the average population of the city [11]. The comparison group did not have a problem with substance abuse as stated by the participants themselves in the questionnaire.

We wanted the comparison group to be larger than the user group in order to increase the power of the statistical tests. A comparison group of twice the size was possible within the limits of this investigation. As we had positive response from all who were asked to participate, the comparison group became a little larger.

On random days the parents of the children under school age were approached by the assistant of the Child Welfare Clinic, and asked if they would be willing to participate in this questionnaire study. In the schools we were present at conferences for the parents some days after written information was sent out. All who had appointments that day were asked to be part of the comparison group. Those who did not keep their appointments had the questionnaire distributed by the teacher and answered at home.

After giving written informed consent to participate in the study, the parents/caregivers of the children from the user group and the comparison group were immediately presented with a questionnaire that was completed on the spot. A person from the project was present to answer any questions the participants might have. The questionnaire included questions about the occurrence of nicotine use and illicit drug use before and during pregnancy, the pregnancy and birth, the current health situation of the mother, and socio-economic issues. The questionnaire was developed by the authors and tested in a pilot.

Data about the user group was also gathered from their medical records at the SCWC. This included self-reported data on substance use before and during pregnancy, results of observed urine tests for substances, and family income and level of education at the start of pregnancy. The urine specimens were analyzed at the laboratory in the local hospital, using tests from Abbot Diagnostics, Axym. The cut off rates were: amphetamine 500 ng/ml, opiates 300 ng/ml, cannabis 50 mg/ml, benzodiazepines 200 ng/ml. We also gathered information about the father's relation to substance use and his contact with the pregnant woman.

In the analyses the users were dichotomized into two groups; "short-term users," including mothers who had stopped the abuse before the end of the first trimester, and "long-term users," including those who continued their substance abuse towards the end of pregnancy.

The data were analysed using bivariate and multivariate techniques in the SPSS-program. The level of significance was set to p ≤ 0.05.

The study protocol was assessed by the Regional Committee for Medical Research Ethics and approved by the Norwegian Data Inspectorate. The study has been conducted in full accordance with the World Medical Association Declaration of Helsinki.

## Results

From the diagnosis register at the local hospital we obtained valid data from the period 1994–2002 on the diagnosis neonatal cramps, cerebral irritation, cerebral depression, reaction on medication, neonatal abstinence syndrome and observation in new borns. During these years one pregnancy did not result in contact with the SCWC. The incidence of children that attended SCWC was 6.1 pr. 1000 births in the county of Vest-Agder (range 4.1–10.2).

Many of the 102 pregnant drug abusers were referred to our clinic by general practitioners (30%). A lot of different initiatives in the community health and social system also contributed, with the substance abuse counselling services referring 25 women (25%), and social/child welfare referring 12 women (12%). The level of education among the users was significantly lower than in the comparison group (See additional file 1). A large percentage had their income from community support. Forty-eight users (47%) had either hepatitis C or B, nine (9%) had both. Treatment for psychiatric disease like depression and anxiety disorders were found in the histories of 36 users (35%), and six (6%) had been diagnosed with somatic diseases like epilepsy, chronic obstructive pulmonary disease, or rheumatism. No user had tuberculosis or HIV. Seven (7%) were homeless. Fifty-two of the women (52%) had been in some sort of treatment for substance abuse and six (6%) were in an institution for treatment for substance abuse when they got pregnant.

Of all the 89 pregnant women that gave birth, only four (4.5%) were unable to cope with the intervention at the SCWC. It was not discovered that one woman continued to use alcohol, one moved to another city where she continued the substance abuse, and two were sentenced to treatment after the new amendment to the Act Relating to Social Services from 1996 [12] where pregnant women that abuse substances can be sentenced to residential treatment until the child is born.

All the 59 pregnant women that were investigated more closely, had a considerable reduction in substance abuse during their pregnancies as established by urine tests and self reports (See additional file 2). Even the 14 percent that had cannabis as their preferred drug had in addition been using great amounts of alcohol or central stimulating drugs like ecstasy or cocaine.

A total of 16 women had older children when admitted to the SCWC for the current pregnancy (22 children were registered). Seven of them had custody of one child each. The children of nine mothers were in foster homes. Twenty-eight (48%) of the 59 women had had adverse sexual experiences, whereof 20 (34%) had experienced both sexual abuse as a child (<16 yrs) and rape as an adult. Twenty-two (37%) women had been convicted of a crime, and nine had served time in prison, mostly short term sentences lasting a few months. One had served a sentence of more than one year in prison.

The women's mean age for starting substance abuse was 15 years (range 11–22). They had been using substances for an average of 10 years (range 2–22) before the current pregnancy. All women abused more than one substance. Of the eight women (14%) that gave cannabis as their preferred drug, all misused alcohol or central stimulating substances as well (See additional file 2). By self report, five pregnant women (8%) had managed to stop all abuse before pregnancy. They were all known by the initiatives cooperating with the clinic and were referred to SCWC for necessary support.

Thirty-six women (61%) agreed to provide supervised urine samples. For 9 women (15%) giving urine samples was not deemed to be necessary, as the user was living with her parents or was cohabitant/married to a man with no drug problems. Fourteen women (24%) refused to provide urine samples. There was a disagreement between the user and the SCWC concerning this, and in these cases there was a great suspicion of continuing substance abuse. Cannabis was the most commonly detected agent among those who tested positive. Seven users had several positive urine tests. Nine women (16%) also stopped smoking when realizing they were pregnant. In general, the partners of the clinic's users had even more serious substance problems than the pregnant woman, except for 15 (24%) men who did not abuse any illicit substances or alcohol. Thirty-one of the women (53%) had contact with the social welfare system during their pregnancy; 25 (42%) with the child welfare service, 26 (44%) with substance abuse counselling, and 29 (32%) with community health services or the hospital. The total number of initiatives in contact with each user is listed in Figure 1.

Univariate analyses of a selection of socio-economic and health variables indicated that members of the short-term user group were less likely than the long-term users to drink alcohol or to smoke during their pregnancy. Still, the drug users were more likely to smoke and use alcohol than the comparison group. More short-term users than long-term users had never been in treatment for substance abuse, and they also had a higher likelihood of having been the victim of rape as an adult. At the same time, fewer in the short-term group received economical support from the community. Both substance abuse in the immediate family and sexual abuse in childhood were more frequent in the user group than in the comparison group. Additional socio-economical and health data are listed in additional file 3.

In a multivariate logistic regression analysis with short/long term drug abuse in pregnancy as a dependent variable, we found that to quit smoking and to be a victim of rape were characteristics significantly associated with stopping drug abuse before the end of the first trimester (See additional file 4).

## Discussion

The main findings in this study are that most of the referred women with substance abuse problems that continued the pregnancy were able to follow a treatment plan at the SCWC, and that we observed a reduction in substance abuse during pregnancy. Extensive collaboration with other services within the community and health system, and home visits if appointments were not kept, contributed to us being able to provide a treatment adjusted for individual needs. This is in agreement with what others have found to be important for pregnant substance abusing women [13-17]. All the users did reduce their abuse of substances during pregnancy as measured by self report and urine tests. The reduction often started before attending the SCWC, which is also consistent with what others have found [18]. Studies have also shown that if pregnant substance abusing women do not attend prenatal care, there is a tendency to continue substance abuse during pregnancy [19].

The group that had experienced rape was significantly more frequent in the group of short-term substance abusers, with an odds ratio of 5.3. Sexual abuse and rape was an important issue to consider when preparing for delivery. Studies have shown that incest survivors might have problems with parenting [20,21]. However, we found no difference in substance abuse during pregnancy among the sexually abused when compared to women without this adverse experience. The pregnancy might be considered as an opportunity to reconstruct a family for women that have abusive partners [22]. As problems resulting from sexual victimisation and the woman's relationship with her partner are important issues when preparing for delivery, it is possible that the intervention at the SCWC had a significant influence on the woman's substance abuse during her pregnancy.

We also found that more women in the short-term group than in the long-term group had previously never been in treatment for their substance abuse, although this difference did not reach significance. Those who had not been in treatment were also significantly younger (p = 0.007) than the group that had been in treatment, and there was a tendency that the pregnant women that had been substance abusers for a short time only, were better able to change their behaviour during pregnancy.

The percentage that stopped using alcohol or tobacco in pregnancy was higher in the short-term group of substance abusers, however compared to the comparison group they smoked and used alcohol more frequently. This finding is supported by other, previous studies [23]. The odds ratio for short-term substance abuse in pregnancy was 9.7 times higher in the group that did not smoke or stopped smoking immediately when they found out they were pregnant. Substance abuse in the immediate family and sexual abuse in childhood was found more often in the drug user group than in the comparison group, which is also what is described by others [24].

As observed in other studies [25], the education level among the substance abusing women was generally low; 88 percent had not finished upper secondary education. The level of education was lower than in the comparison group. Forty-eight percent of the clinic's users were unemployed or in some community supported activity receiving financial support from social security benefits at the time they got pregnant. Commonly, the housing situation for the women was not of a standard where it was possible to raise a child. The group of homeless persons is smaller than found in other studies in Europe [26], but these studies mostly include men. Studies on substance in Scandinavia indicates that the number of homeless people are lower [27].

Among the women that continued the pregnancy, 29 (48%) were positive for hepatitis C or B. This corresponds well with reports for substance abusers from other cities in Europe [26,28]. In this investigation we have not distinguished between intravenous and non intravenous substance abuse, as both can harm the baby before birth and both may destroy the mother-child relationship.

We found that 36 users (35%) had some psychiatric disorder, which is a little lower than reported by others [21,29]. Most studies on psychiatric co-morbidity are from different treatment programs for substance related problems. In our material 52 women (66%) had been in treatment before the pregnancy.

The strength of this study is that we have studied a group of substance abusing, pregnant women in a low-threshold specialised clinic organised within the ordinary primary health care system. The substance abusers had substantial substance problems compared to other studies [25,30]. The incidence of substance abusing pregnant women in the SCWC was 6.1 per 1000 in the years 1994–2002 [31]. The number of substance abusing women differs in different studies [25,32] with lower incidences in studies from Scandinavia. Treatment is only necessary for the women that are addicted, and therefore unable to stop the substance abuse during pregnancy. Our validation based on figures from the diagnosis register in the regional hospital confirms that the majority of women with substance addiction in need of special treatment in this area have been in contact with SCWC.

There are several limitations to this investigation. We have used no tools for assessing the severity of addiction. The substances used before and during pregnancy are registered in the medical records and in the questionnaire. The SCWC is a part of the primary health care system of the community of Kristiansand in which there is little tradition for registering the severity of addiction. Treatment is provided whenever the necessary motivation is obtained, and the registration of addiction in the medical journals is therefore not very detailed. Other studies have shown that information concerning substance abuse given in questionnaires is reliable if there are no other interests influencing the answers given [33,34]. In our study there might have been a fear of legal sanctions. However, in all cases there were persons in the network groups that already knew about the substance abuse, and our experience is that the correct information was given.

The diagnosis of psychiatric disease was done by the patient's regular doctor or a psychiatrist. In the records at the SCWC we only registered self reported psychiatric diagnosis and those who had psychotropic medication during pregnancy. As psychiatric disease changes over time, our registration may not be complete.

In pregnancy the treatment is voluntarily. We were in the beginning anxious that the users of the SCWC would drop out of treatment if we were too determined about urine specimens. Some of the women did not want to give urine specimens, and therefore there might be some uncertainty about their drug abuse in pregnancy.

When investigating the effects of such interventions as those given by the SCWC, it would scientifically have been preferable to have randomised the group in two, where one group had no intervention. Ethically, however, it would be unacceptable to withhold a much needed intervention from a group of pregnant substance abusing women. Our comparison group therefore had to be selected from a normal population. The comparison group consisted of the parents of children coming to their child welfare clinic or coming to parents conferences at school. Both teachers and parents were given information about the project in advance, and the parents also got further information when arriving to the conference. They all expressed a positive attitude to the study. Most parents kept their appointment with the child welfare clinic or school, so very few questionnaires needed to be sent to the homes. When questionnaires were sent to the homes, the appointment was to return the questionnaire with the pupil to the teacher. This might explain why we got answers from all who were asked.

Because of cultural differences it is important to try to identify predictors for reducing substance abuse during pregnancy for substance abusing women in Scandinavia, as the available results in the literature generally are taken from other regions. Future studies would improve on more accurate diagnosis on the level of addiction which can be obtained in closer cooperation with hospitals and treatment institutions for drug abuse, and also there should be a closer follow up by means of urine specimens.

## Conclusion

A low cost and low threshold initiative organised as a child welfare clinic may support substance abusing women in stopping or reducing their substance abuse during pregnancy.

## Abbreviations

SCWC – Special Child Welfare Clinic.

## Competing interests

The author(s) declare that they have no competing interests.

## Authors' contributions

BH conceived of the study and participated in its design, did the statistical analyses and wrote the manuscript. She has also been the doctor at the SCWC since its inception.

ML and EOR participated in the design of the study, helped with statistical analyses and helped draft the manuscript. All authors read and approved the final manuscript.

## Pre-publication history

The pre-publication history for this paper can be accessed here:



## Supplementary Material

Additional file 1 Level of education, employment and economy. Level of education, employment and economy of the 102 users of Special Child Welfare Clinic (SCWC) in Kristiansand, Norway, in the years 1994–2002. Click here for file

Additional file 2 Pattern of substance abuse. Pattern of substance abuse in the users of Special child welfare clinic (SCWC) in Kristiansand, Norway in 1994–2002. N is more than 59 because of polydrug use. Click here for file

Additional file 3 Health and socio-economic issues and their relation to substance abuse during pregnancy. Health and socio-economic issues and their relation to substance abuse during pregnancy among the users of SCWC in Kristiansand, Norway in 1994–2002. Click here for file

Additional file 4 Association between psychosocial variables and the ability to stop substance abuse by the end of the first trimester. Association between psychosocial variables and the ability to stop substance abuse by the end of the first trimester (0 = not stopping, 1 = stop) among the users of SCWC in Kristiansand, Norway, in 1994–2002, from a multivariate logistic regression analysis. Click here for file
