# Supplementary material for: Substance abuse in pregnant women. Experiences from a special child welfare clinic in Norway
Source: BMC Public Health. 2007 Nov 11;7:322. doi: 10.1186/1471-2458-7-322 (PMC2242799; doi:10.1186/1471-2458-7-322)
Supplement: Additional file 1 — Level of education, employment and economy. Level of education, employment and economy of the 102 users of Special Child Welfare Clinic (SCWC) in Kristiansand, Norway, in the years 1994–2002. [file 1471-2458-7-322-S1.pdf]

Table 1. Level of education, employment and economy of the 102 users of Special Child Welfare Clinic (SCWC) in Kristiansand, Norway, in the years 1994-2002.

Special child welfare clinic			Comparison group N=168*		
Education N=94	n	%	n	%	p-value
Not finished lower secondary education (<9 yrs)	10	(11)	0	(0)	0.000
Finished lower secondary education (9 yrs)	58	(62)	19	(11)	0.000
Not finished upper secondary education (<12 yrs)	14	(15)	0	(0)	0.000
Finished upper secondary education (12 yrs)	10	(11)	67	(40)	0.000
College/university	2	(2)	82	(50)	0.000
Employment N=98					
Employed full time	10	(10)			
Employed part time	5	(5)			
Unemployed	36	(37)			
Student	13	(13)			
Home with child	10	(10)			
Disabled	6	(6)			
Community support activity	12	(12)			
Institution patient	6	(6)			
Economy N=96					
Employment	13	(14)			
Student	15	(16)			
Transitional child benefits	10	(10)			
Unemployment benefits	3	(3)			
Rehabilitation benefits	17	(18)			
Social security benefits	38	(40)			

*The educational level of the comparison group was registered in the questionnaire, but has not changed in the years after the child was born.
