# Supplementary material for: Substance abuse in pregnant women. Experiences from a special child welfare clinic in Norway
Source: BMC Public Health. 2007 Nov 11;7:322. doi: 10.1186/1471-2458-7-322 (PMC2242799; doi:10.1186/1471-2458-7-322)
Supplement: Additional file 2 — Pattern of substance abuse. Pattern of substance abuse in the users of Special child welfare clinic (SCWC) in Kristiansand, Norway in 1994–2002. N is more than 59 because of polydrug use. [file 1471-2458-7-322-S2.pdf]

Table 2. Pattern of substance abuse in the users of Special child welfare clinic (SCWC) in Kristiansand, Norway in 1994-2002. N is more than 59 because of polydrug use.

Drugs used before contact with SCWC			Drugs used during pregnancy		Difference		Preferred drug	
N=59	n	(%)	n	(%)	n	(%)	n	(%)
Cannabis	58	(98)	30	(48)	28	(48)	8	(14)
Central stimulating	50	(85)	27	(44)	23	(46)	28	(47)
LSD	14	(24)	0	(0)	14	(100)		
Crack	6	(10)	1	(2)	5	(83)		
Opiates	35	(59)	17	(27)	18	(51)	17	(29)
Sedatives/analgesic/alcohol*	38	(61)	25	(42)	13	(34)	5	(9)
Daily smoking	56	(95)	47	(80)	9	(16)		

*All who abused alcohol also abused sedatives/analgesics.
