# Supplementary material for: Substance abuse in pregnant women. Experiences from a special child welfare clinic in Norway
Source: BMC Public Health. 2007 Nov 11;7:322. doi: 10.1186/1471-2458-7-322 (PMC2242799; doi:10.1186/1471-2458-7-322)
Supplement: Additional file 3 — Health and socio-economic issues and their relation to substance abuse during pregnancy. Health and socio-economic issues and their relation to substance abuse during pregnancy among the users of SCWC in Kristiansand, Norway in 1994–2002. [file 1471-2458-7-322-S3.pdf]

Table 3. Health and socio-economic issues and their relation to substance abuse during pregnancy among the users of SCWC in Kristiansand, Norway in 1994-2002.

Substance abuse in pregnancy	Short time use N=34		Long time use N=25		Total N=59			*Comparison group* N=169		
	n	(%)	n	(%)	n	(%)	p-value	n	(%)	p-value
Substance abuse										
No alcohol pregnancy	23	(68)	9	(36)	32	(54)	0.016	123	(76)	0.001
No smoking in pregnancy	11	(31)	1	(4)	12	(18)	0.008	113	(70)	0.000
Substance abuse<10 years	13	(38)	11	(44)	24	(40)	0.656			
Childs father abuse substances	24	(71)	22	(88)	67	(78)	0.111			
Substance abuse in immediate family	13	(38)	7	(28)	20	(34)	0.412	42	(25)	0.001
Never treated for substance abuse	12	(62)	7	(28)	28	(48)	0.010			
Family/social situation										
Single mother	22	(65)	18	(72)	40	(69	0.554			
Older siblings	23	(68)	20	(80)	43	(73)	0.292			
Community support	12	(35)	16	(64)	28	(48)	0.029			
Convicted	14	(42)	8	(32)	22	(37)	0.471			
Regular at apointments	25	(74)	13	(52)	38	(64)	0.088			
Health										
Prenatal care time less than mean (154 days)	14	(16)	12	(48)	26	(44)	0.602			
Treatment for psychiatric diagnosis*	9	(27)	8	(32)	17	(29)	0.643			
Victim of rape as adult	20	(57)	6	(24)	26	(42)	0.000	7	(4)	0.000
Hepatitis C+B	16	(47)	12	(48)	29	(48)	0.943			
Sexual abuse in childhood	18	(53)	10	(40)	28	(48)	0.325	12	(7)	0.000

*Major depression and anxiety disorder.
