# Supplementary material for: Substance abuse in pregnant women. Experiences from a special child welfare clinic in Norway
Source: BMC Public Health. 2007 Nov 11;7:322. doi: 10.1186/1471-2458-7-322 (PMC2242799; doi:10.1186/1471-2458-7-322)
Supplement: Additional file 4 — Association between psychosocial variables and the ability to stop substance abuse by the end of the first trimester. Association between psychosocial variables and the ability to stop substance abuse by the end of the first trimester (0 = not stopping, 1 = stop) among the users of SCWC in Kristiansand, Norway, in 1994–2002, from a multivariate logistic regression analysis. [file 1471-2458-7-322-S4.pdf]

‘

Table 4. Association between psychosocial variables and the ability to stop substance abuse by the end of the first trimester (0=not stopping, 1=stop) among the users of SCWC in Kristiansand, Norway, in 1994-2002, from a multivariate logistic regression analysis.

		OR (crude)	CI	p-value	OR (adj.)*	CI	p-value
Smoking in pregnancy	Yes (ref)	1.0			1.0		
	No	11.5	(1.4-96.2)	0.024	9.7	(1.1-90.6)	0.045
Treatment for drug addiction	Yes (ref)	1.0			1.0		
	No	4.2	(1.4-12.7)	0.012	3.5	(1.0-12.7)	0.052
Exposed to rape	No (ref)	1.0			1.0		
	Yes	4.0	(1.3-12.6)	0.017	5.3	(1.5-19.0)	0.011
Being regular at appointments	No (ref)	1.0					
	Yes	3.0	(1.0- 9.0)	0.048	Ns		
Use of alcohol in pregnancy	Yes (ref)	1.0					
	No	3.7	(1.3-11.0)	0.018	Ns		

*Adjusted values by forward selection.
